# Extracellular vesicles and cancer stemness in hepatocellular carcinoma – is there a link?

**DOI:** 10.3389/fimmu.2024.1368898

**Published:** 2024-02-27

**Authors:** Lu Tian, Jingyi Lu, Irene Oi-Lin Ng

**Affiliations:** ^1^ Department of Pathology, The University of Hong Kong, Hong Kong, Hong Kong SAR, China; ^2^ State Key Laboratory of Liver Research, The University of Hong Kong, Hong Kong, Hong Kong SAR, China; ^3^ Department of Pathology, The University of Hong Kong-Shenzhen Hospital, Shenzhen, China

**Keywords:** extracellular vesicles, cancer stemness, immune cells, hepatocellular carcinoma, cancer associated fibroblasts (CAF)

## Abstract

Hepatocellular carcinoma (HCC) is a highly aggressive malignancy, with high recurrence rates and notorious resistance to conventional chemotherapy. Cancer stemness refers to the stem-cell-like phenotype of cancer cells and has been recognized to play important roles in different aspects of hepatocarcinogenesis. Small extracellular vesicles (sEVs) are small membranous particles secreted by cells that can transfer bioactive molecules, such as nucleic acids, proteins, lipids, and metabolites, to neighboring or distant cells. Recent studies have highlighted the role of sEVs in modulating different aspects of the cancer stemness properties of HCC. Furthermore, sEVs derived from diverse cellular sources, such as cancer cells, stromal cells, and immune cells, contribute to the maintenance of the cancer stemness phenotype in HCC. Through cargo transfer, specific signaling pathways are activated within the recipient cells, thus promoting the stemness properties. Additionally, sEVs can govern the secretion of growth factors from non-cancer cells to further maintain their stemness features. Clinically, plasma sEVs may hold promise as potential biomarkers for HCC diagnosis and treatment prediction. Understanding the underlying mechanisms by which sEVs promote cancer stemness in HCC is crucial, as targeting sEV-mediated communication may offer novel strategies in treatment and improve patient outcome.

## 1 Introduction

Hepatocellular carcinoma (HCC) is the most prevalent form of primary liver cancers, representing approximately 90% of the cases. It ranks sixth among all malignancies and third among all cancer deaths worldwide (1). The risk factors of HCC are well established and include chronic hepatitis B and C, chronic alcohol consumption, metabolic dysfunction-associated fatty liver disease (MAFLD) (previously named non-alcoholic fatty liver disease [NAFLD]), and cirrhosis of all causes. Notably, the incidences of MAFLD-related HCC have been rising in Western countries in recent years. Unfortunately, HCC is often diagnosed at advanced stages and hence inoperable. Even after operation, there is a high tumor recurrence rate. In addition, HCC is notoriously resistant to conventional chemotherapy. For advanced HCC, the combinational therapy of immune checkpoint inhibitors and anti-vascular endothelial growth factor (anti-VEGF) as well as targeted therapy with tyrosine kinase inhibitors is the first line treatment; however, their therapeutic efficiencies are not satisfactory. Therefore, there is an urgent need for further investigation for better detection methods and more effective treatments for HCC.

Cancer stemness, referring to the stem-cell-like phenotype of cancer cells, has been recognized to play important roles in different aspects of hepatocarcinogenesis. With stem-cell-like or stemness phenotype, the cancer cells possess the ability to regenerate and maintain tumor growth, including tumor initiation, progression, and resistance to chemotherapy (2). Furthermore, metastasis is also a hallmark feature of stemness and involves a series of progressive steps, making it a significant obstacle for effective cancer treatment (3). The Wnt, Notch, Jak, and other pathways are crucial for maintaining stem cell-like traits in tumor cells. Within the tumor microenvironment (TME), neovascular formation and hypoxic conditions enhance the acquisition of stemness features in tumor cells. Additionally, the interaction of immune cells and cancer stem cells (CSCs) facilitates in culturing an immunosuppressive microenvironment. For example, CSCs could polarize the macrophages to tumor associated macrophages (TAMs), which in turn help to maintain the CSC signatures (4).

Extracellular vesicles (EVs) are membrane-bound vesicles that are secreted by cells and can transport nucleic acids, proteins, lipids, and metabolites, hence acting as important mediators of intercellular communication. The two main types of EVs that are extensively studied are exosomes and microvesicles (MVs), which can be distinguished based on their sizes and biogenesis processes. Exosomes, also known as small EVs (sEVs), are typically 30-200 nm in diameter. Their biogenesis begins with the maturation of multivesicular bodies (MVBs), where intraluminal vesicles (ILVs) are generated through cargo sorting. These MVBs are eventually tethered to the plasma membrane and the ILVs are released into the extracellular space. On the other hand, MVs (50-1000 nm in diameter) emerge from the budding of plasma membranes and are directly liberated (5, 6). Since sEVs are distinguished from other types of EVs in their contents and functions and regarded as the principal vehicles that mediate intercellular communication, in this review, we will focus on sEVs or exosomes, excluding other types such as MVs, exomeres, apoptotic bodies, and shed vesicles. Exosomes and sEVs are used interchangeably throughout this article.

**Figure 1:**
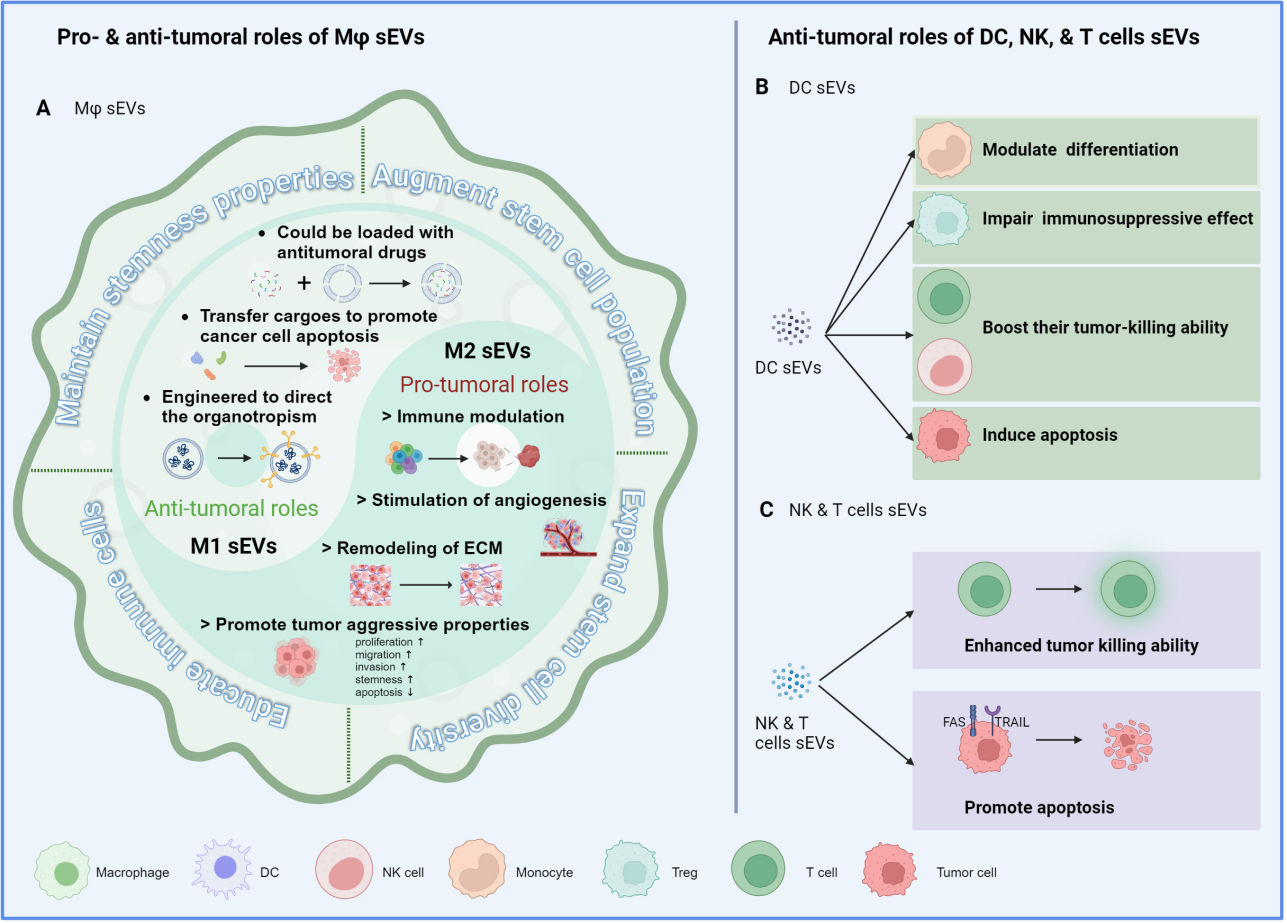
Immune cell-derived sEVs and cancer stemness. **(A)** The mechanisms by which macrophage-derived sEVs modulate the stemness properties of cancer cells, including maintaining their stemness properties, augmenting the stem cell population, expanding the stem cell diversity, and educating the immune cells to an immunosuppressive phenotype. M1-derived sEVs demonstrate antitumoral properties, while M2-derived sEVs exhibit protumoral characteristics, aligning with their parental cells. **(B)** sEVs derived from DCs influence the cancer stemness through regulating the recruitment, polarization, or activation of immune cells. **(C)** sEVs derived from cytotoxic NK and T cells enhance their own tumor killing ability and promote tumor cell apoptosis through ligand-receptor interaction.

**Figure 2:**
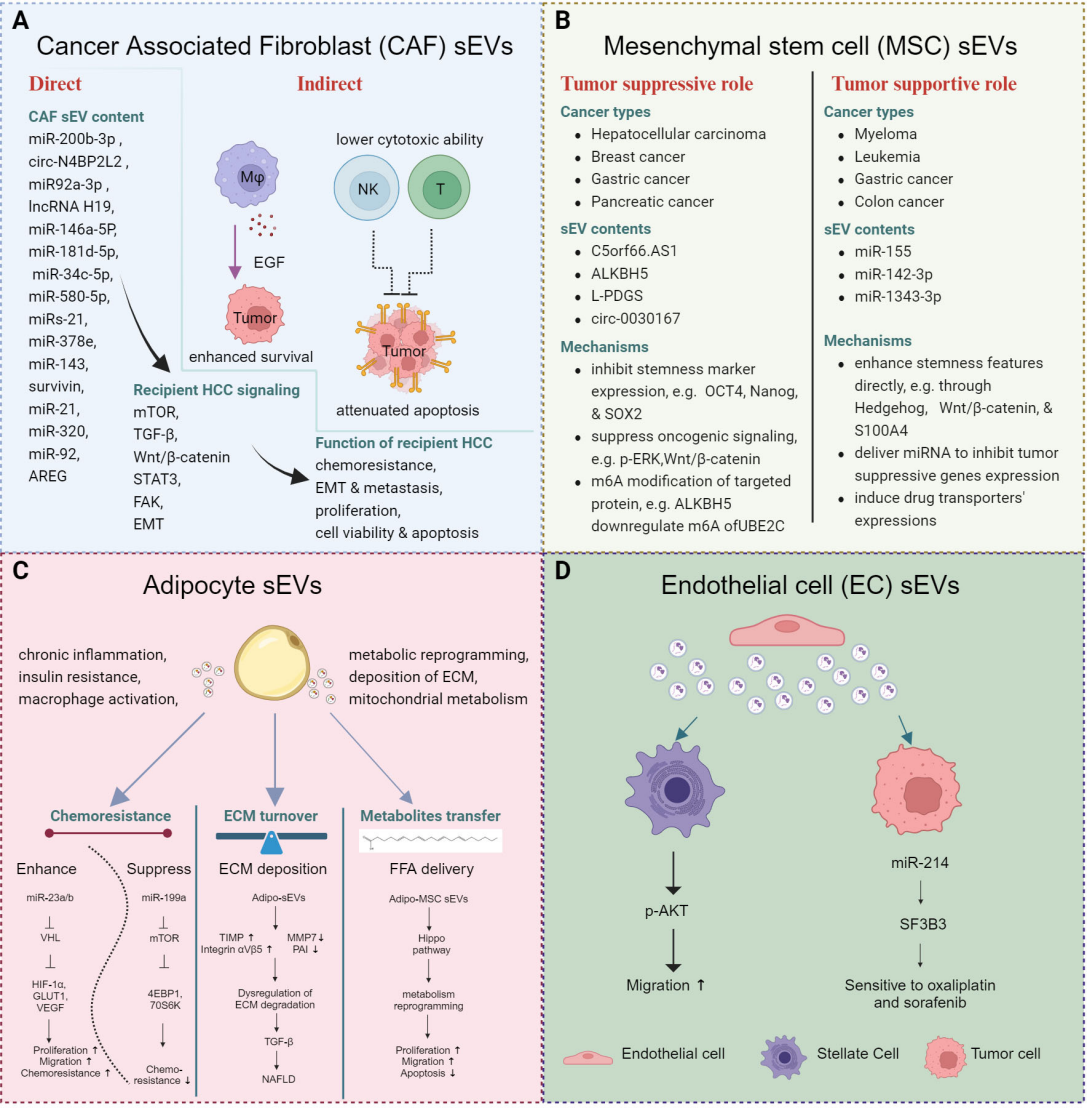
Stomal cell derived sEVs and cancer stemness. **(A)** sEVs derived from CAFs regulate cancer stemness by directly activating tumor cells or indirectly modulating immune cells, ultimately contributing to the prolonged survival of tumor cells. **(B)** sEVs derived from MSCs exert distinct effects in different contexts. **(C)** sEVs derived from adipocytes re-modulate the metabolism of the tumor cells and immune cells and contribute to the development of MAFLD. **(D)** sEVs derived from ECs enhance the migratory ability of hepatic stellate cells and sensitize tumor cells to drug treatment.

**Figure 3:**
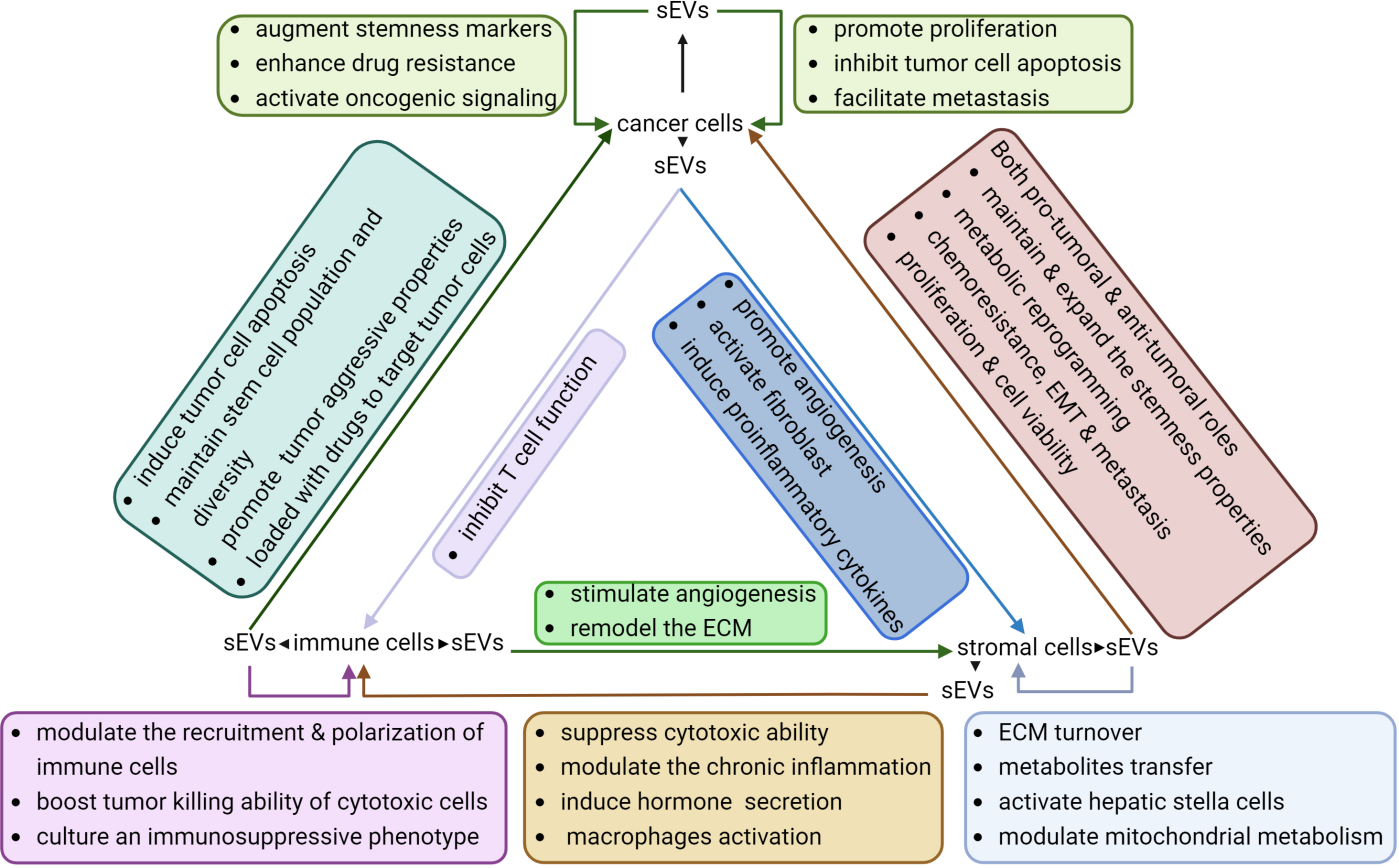
Interactions between sEVs and different cell types within the TME. sEVs derived from tumor cells, immune cells, and stromal cells exhibit diverse effects on their own cells of origin as well as on other cell types. These sEVs play a crucial role in sustaining the stemness of tumor cells by enhancing the aggressive properties of tumors, cultivating an immunosuppressive microenvironment, and facilitating angiogenesis.

In this review, we aim to summarize the current understanding on various aspects of the effects of sEVs on HCC stemness, particularly those secreted from (1) cancer cells (**Table 1**), (2) immune cells (**Figure 1**), and (3) stromal cells (**Figure 2**), as well as the interactions between sEVs and different cell types (**Figure 3**). We will also highlight the potential clinical implications of sEVs in the diagnosis and treatment of HCC. In addition, we will address the limitations of the current sEV-related research and technologies and discuss future research directions that could help overcome these limitations.

**Table 1:** Summary of reports on effects and mechanisms of sEVs from cancer cells on stemness-related features.

Ref.	Exosome Origin	EV content	Functions	Mechanisms
(7)	Spheroid-cultured HCC CSCs	Rab27a	drug resistance and CSC	NANOG
(8)	CD133^+^ HCC CSCs	circZEB1 and circAFAP1	CSC and EMT	E-cadherin, EpCAM, CD90
(9)	CD44^+^ EpCAM^+^ colorectal CSCs	miR-200c	EMT and migration	OCT4, SOX9, NANOG, PI3K/Akt/mTOR
(10)	High-metastatic HCC cell lines	CPE	proliferation and migration	cyclin D1, c-myc
(11)	High-metastatic HCC cell lines	S100A4	CSC and migration	Stat3, osteopontin
(12)	High-metastatic HCC cell lines	TMPRSS2, NID	proliferation and migration	NID
(13)	HCC cell lines	miR-4800-3p	CSC and EMT	CD44, CD133, Oct4, E-cadherin, ZO-1, STK25p-YAP, TAZ, PCNA
(14)	HCC patient serum	pIgR	CSC and migration	PDK1, Akt, GSK3b, b-catenin
(15)	HCC cell lines	slug	CSC and EMT	CD133, FN1, COL2A1
(16)	HCC cell lines	S100A10	drug resistance, CSC, proliferation, and migration	MMP2, EGF, fibronectin, ITGAV
(17)	HCC cell lines	circCCAR1	drug resistance	PD1
(18)	Sorafenib resistant HCC cells	miR-744	drug resistance and proliferation	PAX2
(19)	HCC cell lines	miR-3129	proliferation and migration	N-cadherin, E-cadherin, TXNIP
(20)	HCC cell lines	circ_002136	apoptosis	miR-19a-3p, RAB1A
(21)	HCC cell lines	DDX55	proliferation, migration, and angiogenesis	BRD4, PI3K, p-Akt, b-catenin, p-GSK3b
(22)	HCC cell lines	LINC00161	proliferation, angiogenesis and migration	miR-590-3p, ROCK2, MMP-2, MMP-9, VEGFA
(23)	HCC cell lines	miR-25	proliferation, apoptosis, CSC, and migration	SIK1
(24)	HCC cell lines	CTLA	proliferation, drug resistance, and migration	CAPG
(25)	HCC cell lines	Rab27a	EMT, migration, and proliferation	N-cadherin, vimentin, XEB2, OVOL1, Slug, p-Erk1/2
(26)	HCC cell lines	circGSE1	EMT, migration, and drug resistance	miR-423-5p, TGFBR1, SMAD3
(27)	HCC and mammary adenocarcinoma cells	Rab27a	EMT, migration, and proliferation	Twist, Slug, vimentin, N-cadherin, VEGF, TGFb
(28)	HCC cell lines	ENO1	EMT, migration, and proliferation	E-cadherin, Vimentin, FAK/Src/MAPK
(29)	Adenocarcinoma and HCC cell lines	LOXL4	EMT and migration	p-FAK, p-Src
(30)	HCC cell lines	ST6Gal-I	EMT and migration	MMP2, MMP9, p-Smad2, p-Smad3, p-AKT, p-GSK, p-catenin, p-ERK1/2, p-JNK, p-P38, p-NF-kB
(31)	HCC tissues	circTTLL5	EMT, migration, and proliferation	miR-136-5p, KIAA1522
(32)	HCC cell lines	circPTGR1	EMT and migration	CCND1, CDK4, MET, miR-449a
(33)	HCC cell lines	lnc_SNHG16	angiogenesis, migration, and proliferation	PI3K/Akt/mTOR
(34)	HCC cell lines	miR-584-5p	angiogenesis, EMT, and migration	VEGF, VEGFA, MMP-2, MMP-9, PCK1, Nrf2
(35)	HCC cell lines	14-3-3ζ	TME and drug resistance	PD1
(36)	Hypoxic HCC cell lines	miR-155	angiogenesis	Not explored
(37)	HCC cell lines	miR-155	proliferation	PTEN
(38)	Hypoxic HCC cell lines	miR-1273f	proliferation and migration	E-cadherin, miR-221-3p, miR-93-5p
(39)	Hypoxic colorectal cancer cell lines	miR-361-3p	Proliferation and apoptosis	TRAF3, NF-kB

## 2 sEVs derived from HCC cancer cells and cancer stemness

In general, sEVs derived from either HCC cells or HCC CSCs were found to promote various CSC features of recipient HCC cells or non-cancer cells. At the beginning of this section, we would like to highlight the few studies that particularly investigated the distinctive or additional effects of sEVs derived from CSC HCC cells. Huang et al. demonstrated that sEVs derived from spheroid cultures promoted regorafenib resistance of various recipient non-CSC HCC cell lines as compared to adherent cultures. Specifically, RAB27A upregulation in CSC-derived sEVs induced Nanog expression in recipient non-CSC HCC cells (7). Besides, Han et al. sorted CD133^+^ HepG2 and Huh7 cells as representative of CSC HCCs. The sEVs derived from these CD133^+^ HCCs, as compared to the sEVs secreted by the CD133^-^ cells, were shown to promote migration, proliferation, self-renewal, and epithelial-mesenchymal transition (EMT) of target non-CSC HCC cells *in vitro*, via transfer of circ-ZEB1 and circ-AFAP1 (8). Similar to HCC CSCs, CD44^+^ EpCAM^+^ colorectal CSCs secreted exosomes that were enriched in miR-200c, resulting in subsequent activation of PI3K/Akt/mTOR signaling in target cells, which suggested that the metastatic properties could be transferred from highly to lowly metastatic cells via sEVs (9). sEVs from highly metastatic cell lines were found to be enriched in carboxypeptidase E (CPE) and S100A4 and promote the proliferation and metastatic potential of non-CSC HCC cells, through activating c-myc/cyclin D1 and STAT3/OPN signaling, respectively, in the recipient cells (10, 11). Moreover, highly metastatic HCC cells were also found to secrete sEVs that were deprived of transmembrane serine protease TMPRSS2; those sEVs were found to suppress the packaging of pro-tumoral nidogen into exosomes and inhibit proliferation and migration of immortalized hepatic cell LO2 (12).

In this section, we will summarize the various contents of exosomes that have been identified to promote cancer stemness. Additionally, we will discuss the different features of CSCs that can be regulated by cancer cell-derived sEVs, as well as the underlying mechanisms involved in these processes.

### 2.1 sEVs from cancer cells upregulate the expression levels of stemness markers

Upregulation of relevant CSC markers is one of the key features that represent the enhanced stemness properties. Several studies identified specific exosomal proteins and miRNAs that induced the expression of CSC markers in HCC. For instance, miR-4800-3p was found to be overexpressed in exosomes derived from Huh7 cells treated with TGF-β, as well as in the exosomes from HCC patients’ blood. This upregulation of miR-4800-3p was associated with enhanced stemness potential and upregulation of CSC markers, including CD44, CD133, and OCT4 (13). Another study revealed that the polymeric immunoglobulin receptor (pIgR) was elevated in the plasma-derived sEVs from late-stage HCC patients compared to those from early-stage, cirrhotic, HBV positive patients or healthy subjects. Importantly, elevation of pIgR was associated with metastasis, and treatment with these sEVs led to a significant upregulation of CSC markers, including CD90 and CD133 (14). Furthermore, exosomes from Slug-overexpressing HCC cells were found to increase CD133 expression in recipient cells compared to control exosomes (15). These findings highlight the role of specific exosomal miRNAs and proteins in promoting CSC marker expressions and enhancing stemness properties of HCC.

### 2.2 sEVs from cancer cells enhance drug resistance

CSCs are resistant to chemotherapies and radiotherapies due to higher levels of drug efflux transporters and dysregulated apoptotic pathways. Many exosomal cargos were reported to promote HCC drug resistance through regulating the stemness potential. Among those, proteins located on the surface of cancer cell-derived sEVs were research hotspots on drug resistance, as their functions could be blocked by neutralizing antibody or small molecule inhibitors, therefore providing potential clinical application. HCC cell-derived sEVs containing S100 calcium binding protein A10 (S100A10) was found to facilitate HCC progression through promoting multifaceted functions including stemness validated by enhanced sphere formation. Neutralizing antibody targeting sEV-S100A10 was able to sensitize HCC cells to sorafenib treatment, and combination treatment synergistically reduced tumor growth in subcutaneous xenograft model. The neutralizing antibody also suppressed metastasis in intrasplenic and tail-vein injection models (16). Besides proteins, circular RNAs or miRNAs within cancer cell-derived exosomes were also found to promote drug resistance of HCC cells. Specifically, tumoral exosomal circCCAR1 level was significantly and negatively correlated with the number of infiltrating CD8^+^T cells, and the exosomal circCCAR1 could inhibit the cytotoxicity of CD8^+^ T cells while suppressing PD1 degradation. These findings suggest a potential mechanism behind HCC resistance to anti-PD1 therapy (17). Additionally, exosomes secreted by sorafenib-resistant HCC cells were found to contain lower levels of miR-744 than non-resistant HepG2 cells. When treated with miR-744-enriched exosomes, HCC cells exhibited a reduction in proliferation (18). Treatment with HCC sEVs activated multiple molecules that were associated with enhanced drug resistance. These molecules included p-AKT, p-GSK3β, EMT, matrix metalloproteinases (MMP), and p-STAT3 (11, 14, 16). These findings emphasized that sEVs derived from tumor cells could enhance drug resistance of recipient cells through multiple signaling pathways.

### 2.3 sEVs from cancer cells dysregulate proliferative and apoptotic abilities of recipient HCC cells

sEVs derived from HCC cells were found to promote proliferation and inhibit apoptosis in the recipient cells of different HCC cell lines. This phenotypic change could be mediated by the transfer of miR-3129 through sEVs, which targeted TXNIP (19). Furthermore, transfer of exosomal Circ_002136 was found to inhibit apoptosis in HCC cells. Circ_002136 inhibited miR-19a-3p, thus relieving its suppression on the miRNA target, Ras-related protein Rab-1A (RAB1A) (20). In another study, highly metastatic HCC cells, as compared to lowly metastatic cells, were shown to secrete exosomes with a lower level of TMPRSS2, a transmembrane serine protease. TMPRSS2-enriched exosomes were found to suppress the packaging of pro-tumoral nidogen into exosomes and inhibit proliferation and migration of immortalized hepatic cell (12). In addition, various molecules, including DEAD-box helicase 55 (DDX55), CPE, miR-3129, LINC00161, clathrin light chain A (CTLA), and miR-25, were found to be enriched in cancer-derived exosomes and induce cell proliferation (10, 19, 21–24). Signaling alterations upon sEV treatment primarily involved EMT markers, Wnt/β-catenin signaling, and cell cycle pathways (19, 21, 23). These findings suggest potential mechanisms behind the crosstalk between drug resistance pathways and proliferation signaling via sEVs in HCC.

### 2.4 sEVs from cancer cells promote metastasis

Enhanced metastatic ability is one of the stemness features. HCC cell-derived exosomes have been found to generally promote EMT and migration in both recipient HCC cells (25, 26) and endothelial cells (27). Treatment of HCC cells with exosomes derived from enolase 1 (ENO1)-overexpressing HCC cells resulted in higher levels of E-cadherin and vimentin, and lower levels of N-cadherin. Additionally, treatment with ENO1-overexpressed exosomes induced the activation of integrin α6, integrin β4, phosphorylated p38, phosphorylated FAK, and phosphorylated Src, indicating the activation of the FAK/MAPK pathway and the induction of metastatic potential (28). Similarly, exosomes derived from lysyl oxidase homolog 4(LOXL4)-enriched HCC cells were found to promote EMT and migration in recipient HCC cells through the activation of the FAK/MAPK pathway (29). α2,6-sialyltransferase I (ST6Gal-I), an important mediator in the exosome biogenesis pathway, was found to play a critical role in activating AKT/GSK and JNK1/2 signaling in the recipient cells, as well as inducing MMP2 and MMP9 secretion, thereby promoting proliferation and migration (30). Besides proteins, circular RNAs packed within the exosomes were also found to regulate EMT signaling within the recipient cells. Liu et al. isolated exosomes from HCC tumor samples and identified circTTLL5 to be significantly upregulated in tumor-derived as well as serum-derived exosomes in HCC patients. Mechanistically, circTTLL5 was found to sponge and inhibit miR-136-5p. This inhibition activated pro-metastatic KIAA1522, led to upregulation of EMT markers and induced proliferation (31). Similarly, exosomal circPTGR1 was shown to promote EMT of target HCC cells and increase the number of metastatic lymph nodes *in vivo*, through suppressing miR-449 (32).

### 2.5 sEVs from cancer cells modulate tumor microenvironment

In addition to directly regulating the behavior of tumor cells, tumor exosomes can also regulate the activation of stromal cells and immune response. One study found that the lncRNA small nucleolar RNA host gene 16 (lnc-SNHG16) activated the PI3K/AKT/mTOR pathways in HUVEC cells by sequestering miR-4500 and releasing the expression of polypeptide N-acetylgalactosaminyltransferase 1 (GALNT1). This led to increased angiogenesis and proliferation of HUVEC cells (33). Furthermore, another study demonstrated that exosomal miR-584-5p promoted HCC angiogenesis by being taken up by endothelial cells and suppressing PCK1-mediated NRFG2 activation (34). In addition to endothelial cells, fibroblasts can also be activated when exposed to sEVs derived from HCC cells. Specifically, the tumor sEVs carried decreased levels of TMPRSS2, which led to the activation of fibroblasts through the phosphorylation of p65 and increased α-SMA expression. This activation was accompanied by the production of higher levels of proinflammatory cytokines, ultimately enhancing the proliferation, migration, and colony formation abilities of fibroblasts (12). In some cases, tumor sEVs did not directly affect tumor cells or stromal cells but instead regulated the function of immune cells. For instance, exosomes carrying 14-3-3ζ could be taken up by T cells and thus inhibited their proliferation, activation, and anti-tumor functions. In addition, exosomal 14-3-3ζ could induce PD1 expression on T cells, potentially leading to resistance towards PD1/PD-L1 therapy (35). Therefore, not only the stemness properties could directly be regulated by cancer-derived sEVs, but could also be enhanced through the activation of fibroblasts or suppression of immune responses.

### 2.6 sEVs from hypoxic cancer cells promote tumor growth

Because of its fast-growing property, the TME of HCC is frequently hypoxic. sEVs secreted under hypoxia generally displayed supporting roles in HCC. For example, miR-155 was often enriched in exosomes secreted by HCC cells under hypoxic condition. Administration of miR-155-enriched exosomes to HUVEC cells could induce tube formation and potentially facilitate HCC metastasis (36). Furthermore, administration of exosomal miR-155 could directly suppress the PTEN expression of HCC cells, thus promoting proliferation and tumor growth (37). Similar to miR-155, miR-1273f was upregulated in hypoxia-induced exosomes, and it could suppress the expression of LHX6, an inhibitor of the Wnt/β-catenin pathway (38). In addition to HCC, hypoxia was able to induce the expression of miR-361-3p in colorectal cancer cell-derived sEVs, which facilitated tumor growth and suppressed the cell apoptosis through NF-κB pathway (39).

## 3 sEVs from immune cells and HCC stemness

Immune cells make up a significant proportion of a tumor mass. They either originate from the primary organ or are recruited from circulating bone marrow cells. As the first line of defense, innate immune cells, such as macrophages, neutrophils, dendritic cells (DCs), and natural killer (NK) cells, induce nonspecific, quick immune responses to eliminate tumor cells. In addition, adaptive immune cells, mainly T and B cells, can elicit humoral and cellular immunity to specifically destroy tumor cells. However, during tumor progression, the roles of immune cells may shift from anti-tumoral to pro-tumoral, and various immune components exert synergistic or disparate functions based on their characteristics. In this section, we will provide a summary of how immune cell-derived sEVs influence the behavior of tumor cells, with a particular focus on HCC (**Figure 1**).

### 3.1 Macrophage-derived sEVs

Macrophage-derived sEVs play a complex role in tumors and have both pro-tumorigenic and anti-tumorigenic effects, depending on whether they originate from M1 (anti-tumorigenic) or M2 (pro-tumorigenic) macrophages. Furthermore, sEVs derived from M1 macrophages (M1-sEVs) can be utilized as therapeutic vehicles loaded with anti-tumoral chemical drugs or specific antineoplastic proteins to enhance tumor cell apoptosis at the tumor site. On the other hand, the genetic materials present in M2-sEVs, such as miRNAs, lncRNAs, and circRNAs, contribute to the recruitment, polarization, and metabolic reprogramming of macrophages to support tumor development. Notably, macrophage-derived sEVs can facilitate cancer cells in maintaining their stemness properties and augmenting the stem cell population and diversity. Additionally, these sEVs are able to educate immune cells to adopt an immunosuppressive phenotype, thus facilitating the survival and expansion of CSCs. In HCC, sEVs derived from tumor-associated macrophages (TAM-sEVs) within HCC tissues can transfer enhanced proliferative potential, migratory ability, and sphere-forming capacities, as compared to sEVs derived from macrophages in the peritumoral tissues. These effects were attributed to the downregulation of miR-125a and miR-125b, which relieved the suppression of the stemness marker CD90 in HCC cells (40). Zhang et al. demonstrated that overexpression of RBPJ in macrophages resulted in the upregulation of circ_0004658, both in the parental cells and the secreted EVs. Functionally, these EVs suppressed cell proliferation and promoted apoptosis by abolishing the suppression of miR-499b-5p towards JAM3 (41). In addition to the reported function of sEVs from pan macrophages, it was found that M1- and M2-sEVs exhibited distinct functions towards HCC cells. Specifically, M1-sEVs upregulated miR-326, leading to HCC cell apoptosis and cell cycle arrest via the NF-κB pathway (42). M2-sEVs with overexpressed miR-660-5p were observed to enhance cell stemness in HepG2 cells by suppressing Kruppel-like factor 3 (KLF3) (43). Moreover, transfer of miR-17-92 clusters from M2-sEVs to tumor cells was found to stimulate an imbalance of the TGF-b1/BMP-7 pathways in HCC cells, thereby facilitating EMT and stemness potential (44).

### 3.2 Dendritic cell-derived sEVs

Studies have shown that sEVs derived from DCs (DC-sEVs) carry the functional MHC-peptide complexes and immune-stimulating molecules. DC-sEVs mainly modulate the differentiation of monocytes, boost the tumor-killing ability of T cells and NK cells, and impair the immunosuppressive effect of Treg cells (45). In HCC, exosomes derived from AFP-expressing DCs triggered potent antigen-specific immune responses leading to substantial inhibition of tumor growth, and improved the survival rates of mice. This effect was mediated by an increase of IFN-γ-expressing CD8^+^ T lymphocytes, accompanied with higher levels of IFN-γ and IL-2 production, and a decrease of CD25^+^ Foxp3^+^ regulatory T cells with reduced levels of IL-10 and TGF-β in xenograft tumors (46). Moreover, DC-sEVs can act on both tumor cells and NK cells to exert anti-tumor roles. For instance, DC-sEVs expressed TNF, FasL, and TRAIL on their surfaces, enabling them to induce cancer cell apoptosis through caspase signaling in tumor cells. Simultaneously, DC-sEVs activated NK cells and stimulated the secretion of IFN-γ (47). Notably, DC-sEV-based vaccines have been one of most promising and widely used cancer immunotherapies (48). The therapeutic potential of DCs will be further discussed in the section on clinical implications.

### 3.3 Neutrophil-derived sEVs

The role of neutrophils in tumor progression has been a topic of debate, and their plasticity has been suggested to explain their dual roles in the TME. Neutrophils possess the ability to interact with other immune cells, including macrophages, lymphocytes and NK cells, and this interaction is recognized as a critical aspect of their biology. Neutrophil-derived sEVs (N-sEVs) are distinct from others since they are synthesized and secreted on-demand and thus their compositions are customized for the specific pathological conditions. Similar to macrophages, neutrophils are generally classified as inflammatory N1 neutrophils or N2 neutrophils, which are involved in tissue regeneration and angiogenesis and are especially important for supporting tumor growth. Overall, N1-sEVs can induce anti-tumor effects and augment immune responses against tumors. In addition, N1-sEVs contain several other factors that play a role in cell migration, including 5-lipoxygenase-activating protein (FLAP), 5-lipoxygenase (5-LO), and leukotriene B4 (LTB4) (49). Conversely, N2-sEVs contain high levels of myeloperoxidase and have been linked to tumor progression (50, 51). To date, there is still a lack of studies regarding the functions of N-sEVs in HCC.

### 3.4 NK and T cell-derived sEVs

sEVs from cytotoxic cells, such as NK and T cells, inherit the antitumor properties from their parental cells and can induce tumor cell apoptosis either directly or via immunomodulation. For instance, Vδ2-T derived exosomes promoted the apoptosis of EBV-associated tumor cells and stimulated CD4 and CD8 T cell-mediated antitumor immunity (52). Besides, NK-sEVs exhibited a higher efficiency in targeting tumor cells through natural cytotoxicity receptors (53). For instance, Fas-L was detected on the surface of NK-sEVs, and the binding of Fas-L to FAS or TRAIL on the tumor cell surface could trigger caspase-dependent apoptosis (54). Moreover, DNAX accessory molecule-1 (DNAM1), present in NK-sEVs, enhanced its uptake and cytotoxicity within target tumor cells. Blocking DNAM1 using antibodies delayed NK-EV-mediated apoptosis of tumor cells (55). Similar to DC-sEVs, NK-sEVs are promising therapeutic vehicles under extensive research.

## 4 sEVs from stromal cells and HCC stemness

Stromal cells encompass a diverse population, including cancer associated fibroblasts (CAFs), mesenchymal stem cells (MSCs), adipocytes, and endothelial cells. These cells play crucial roles in shaping the TME and influencing tumor progression (**Figure 2**).

### 4.1 Cancer associated fibroblast-derived sEVs

CAF-sEVs interact with various cell types, including tumor cells, stem cells, endothelial cells, fibroblasts, and macrophages. These sEVs can modulate the behavior of tumor cells directly or educate immune cells to generate immunosuppressive environments to support tumor progression in a comprehensive manner.

In HCC, CAF-sEVs could enhance tumor aggressive features, including enhanced proliferation, augmented invasive potential, and heightened stemness properties. In regulating the tumor cell behavior, CAF-sEVs were found to induce, maintain, and expand the stemness properties in various cancers. Several miRNAs and circRNAs were reported to be transferred from CAFs to other cells through sEVs and activate corresponding signaling to induce cellular phenotypic changes. For example, miR-200b-3p (56), circ N4BP2L2 (57), miR92a-3p (58), and lncRNA H19 (59), which were transferred from CAFs to the colon cancer cell cells via sEVs, were able to regulate tumor properties, including stemness, chemoresistance, metastasis, and EMT changes. Moreover, several components derived from CAF-sEVs are highlighted for driving stemness or chemoresistance, including miR-146a-5P in bladder cancer (60); miR-181d-5p in renal cell carcinoma (61); miR-34c-5p in laryngeal cancer (62); miR-580-5p (63), miRs-21, -378e and -143 (64), survivin (65) in breast cancer; and miR-21 in oral cancer (66). Regarding HCC, one study demonstrated that the loss of miR-320 in CAF-sEVs activated the ERK1/2 signaling through PBX3 to facilitate HCC progression (67). Interestingly, CAF-sEVs were also reported to contribute to the survival of HCC cells through macrophages. Miyazoe et al. demonstrated that the sEVs from senescent hepatic stellate cells (HSCs) did not affect the secretion of growth factors of hepatoma cells. Instead, macrophages treated with HSC-sEVs showed EGF upregulation. Subsequently, the sEV-treated macrophages promoted HCC cell survival (68). The CAF-sEVs induced not only aggressive properties of tumor cells, but they also primed an immune-suppressive TME. Dou et al. demonstrated that the upregulated miR-92 in CAF-sEVs promoted tumor cell growth and migration as well as suppressed the cytotoxic function of NK cells and T cells, via upregulating the PD-L1 expression on breast cancer cells. A suppressed target, LATS2, further activated the YAP nuclear translocation to contribute to the upregulation of PD-L1 (69). These findings pinpointed a more complicated interaction and feedback network among stromal cells, immune cells, and tumor cells. Of significance, mTOR, TGF-β and Wnt/β-catenin signaling were identified to be associated with CAF-sEVs functions across different cancer types (57, 58, 60, 61, 70).

### 4.2 Mesenchymal stem cell-derived sEVs

MSCs display multi-lineage differentiation potential. Likewise, depending on the specific cancer type, MSC-sEVs exhibit dual roles, both supportive and suppressive. Through the transfer of cargos directly to recipient cells or by interacting with ligand-receptor pairs, they activate various signaling pathways. MSC-sEVs were reported to primarily regulate stemness features in target cells. With MSC-sEV treatment, lncRNA C5orf66.AS1 was found to be upregulated, with consequential elevated expression of dual specificity phosphatase 1 (DUSP-1) and suppression of p-ERK, which resulted in the inhibition of malignant behavior of HCC CSCs (71). MSC-sEVs also showed tumor supporting roles in other several cancer types. For example, sEVs from BM-MSCs promoted the chemoresistance of leukemia cells through upregulating S100A4 (72). Contradictory results were observed in other cancer types, in which MSC sEVs had the potential to be used as therapeutic strategy to suppress tumor progression. M6A regulator AlkB homolog 5 (ALKBH5) could suppress the stemness and metastasis of triple-negative breast cancer (TNBC) through modulating the m6A modification of UBE2C cells and reducing p53 expression. ALKBH5 could be loaded within sEVs and exert a tumor suppressive role (73). In addition, exosomal circ-0030167 suppressed the stemness of pancreatic cancer cells through inhibiting miR-338-5p and targeting the Wnt/β-catenin axis (74). These data pinpoint that MSCs may hold promise for a wide range of applications in the treatment of cancer.

### 4.3 Adipocyte-derived sEVs

MAFLD may develop into cirrhosis or even HCC; however, the underlying mechanism of how adipocytes affect the biological behavior of hepatocytes and confer the stemness and aggressive potential to HCC cells is unclear. Recently, emerging evidence has unveiled that the sEVs aid the communication between adipocytes and hepatocytes or HCC cells. Adipocyte-derived sEVs are mainly reported to modulate chronic inflammation, insulin resistance, macrophage activation, metabolic reprogramming, and deposition of extracellular matrix (ECM) with subsequent fibrosis or MAFLD. The visceral adipose tissue (VAT)-derived EVs from obese patients, but not the lean donors, could maintain the ECM turnover through upregulating TIMP-1 and downregulated MMP7 and plasminogen activator inhibitor 1 (PAI) (75). Moreover, it could upregulate the integrin ɑVβ5 to activate TGF-β signaling with consequent NAFLD. Besides, adipocyte-derived sEVs also facilitated HCC development through transferring miR-23/b to HCC cells (76). Moreover, the sEVs derived from adipocyte MSC (AMSC-EVs) were able to transfer miR-199a to HCC cells, sensitizing them to doxorubicin treatment (77). This was achieved by suppressing mTOR signaling, as evidenced by decreased expression levels of mTOR and phosphorylated 4EBP1 and 70S6K in HCC cells.

### 4.4 Endothelial cell-derived sEVs

Few studies on the role of exosomes from endothelial cells (ECs) have been reported. Want et al. found that exosomal SK1 from ECs enhanced HSC migration, accompanied with upregulation of p-AKT (78). Additionally, sEVs from human cerebral ECs (hCEC) carrying abundant miR-214 could sensitize HCC cells towards oxaliplatin and sorafenib treatment through inhibition of splicing factor 3B subunit 3 (SF3B3) expression (79).

## 5 Clinical implications and therapeutic opportunities

### 5.1 Diagnostic and predictive values of sEVs

Current methods for HCC diagnosis involve utilizing plasma markers, such as alpha-fetoprotein (AFP) and des-gamma-carboxy prothrombin (DCP), in combination with imaging techniques. sEVs have emerged as promising biomarkers for HCC due to their higher levels of expression of candidate markers, non-invasiveness, and higher marker stability. Potential prognostic biomarkers, such as miR-21 (80), are usually selectively enriched in sEVs during sEV biogenesis. Moreover, sEVs can be isolated from various body fluids, such as blood or urine, allowing non-invasive detection and monitoring. In addition, thanks to the unique membrane structure of sEVs, cargos are protected from degradation by RNAse or proteinase. As a result, as compared to cell-free DNAs, sEVs-DNAs exhibited an enhanced stability, resulting in a greater detectability of their mutations (81).

Plasma sEVs have been implicated in the diagnosis of HCC through various approaches, such as individual cargo analysis, combined analysis of multiple sEV cargos, or in conjunction with traditional biomarkers. As a single marker, exosomal miR-10b-5p demonstrated the ability to distinguish early-stage HCC patients from healthy individuals, achieving an impressive area under the ROC curve (AUC) value of 0.934 (82). Through combining multiple sEV cargos, an HCC EV ECG score, which is an EV-based protein assay and calculated from the readouts of three HCC EV subpopulations (EpCAM^+^CD63^+^, CD147^+^CD63^+^, and GPC3^+^CD63^+^ EVs), was established for detecting early-stage HCC (83). Moreover, the combination of four exosomal miRNAs (miR-10b-5p, miR-21-5p, miR-221-3p, and miR-223-3p) was significantly effective in differentiating low-AFP HCC from other liver diseases with an AUC of 0.80 (84). In conjunction with traditional markers, the combination of miR-122, miR-148a and AFP gained the highest AUC (0.990) in differentiating HCC from normal controls (85). Besides, AFP together with the levels of HCC-associated EV markers, such as miR-483-5p or AnnV^+^ CD44v6^+^, was found to significantly raise the sensitivity and specificity of HCC diagnosis (86, 87).

For detecting AFP-negative HCC, it has been shown that of the five plasma sEV-miRNAs that were upregulated in HCC, miR-19-3p demonstrated the most effective diagnostic performance and had a high sensitivity (88). Compared to chronic HBV and liver cirrhosis, circ_0028861 exhibited downregulation in HCC patients and showed greater significance than AFP in diagnosing small, early-stage, and AFP-negative HCC (89). Through profiling lncRNA from plasma exosomes, the expression level of exosomal RP11-85G21.1 demonstrated its effectiveness as a biomarker for AFP-negative HCC (90).

sEVs have been utilized not only as diagnostic markers, but they also as predictive indicators for treatment response and prognosis prediction. For instance, the reduced levels of miR-125b in sEVs was reported to predict a higher recurrence rate after surgery in HCC (91). Another study showed that higher levels of miR-320d in serum EVs were linked to better recovery in HCC patients after surgery (92). Moreover, the expression of plasma exosomal miR-4669 could predict HCC recurrence after liver transplantation with specificity and sensitivity at around 75% (93). The expression of exosomal miR-122 was found to be significantly reduced post transarterial chemoembolization (TACE), and the ratio of serum exosomal miR-122 post TACE as compared to pre-TACE was positively correlated with prolonged disease-specific survival, indicating its potential clinical utility (94). Additionally, the level of exosomal circ-G004213 can serve as a predictor of cisplatin resistance in HCC (95).

In summary, EV cargos, encapsulated with a significant portion of cancer-related attributes, have the potential to be used as liquid biopsy markers to monitor treatment response and predict prognosis in HCC.

### 5.2 Therapeutic implications of sEVs

As mentioned in previous sections, sEVs have emerged as a potential vehicle for drug delivery in cancer therapy as well as neuron disease (96), with their advantages in solubility, stability, specificity, and biocompatibility. sEVs originating from specific cells, like mesenchymal stem cells and immune cells, hold promise for clinical therapy since they carry traits similar to those of their parent cells (97). sEV-based therapy involves altering EV contents, or blocking EV-mediated pro-tumoral functions. Compared to small molecular chemical drugs, especially hydrophobic drugs, nano-size sEVs with membrane structure showed much higher solubility and effectively penetrate biological barriers (blood-tumor barrier, blood-brain barrier, blood-marrow barrier, gastrointestinal barrier, etc.) (98–100). Based on the findings that exosomal integrins (ɑ6β4 and ɑ6β1) could direct tumor metastasis to the lung, while exosomal integrin ɑVβ5 was associated with liver metastasis (101), manipulation of the surface proteins such as integrins of sEVs could alter their organ tropism and hence delivery of anti-cancer drugs or nucleic acids by sEVs to target organs. In addition, compared to some naked RNA such as siRNAs, RNAs loaded in sEVs could protect them from degradation and maintain their high stability.

In engineering sEVs, the chemical drugs or nucleic acids could be loaded into sEVs directly, by transfection, electroporation, and saponin-assisted methods, or indirectly by manipulation of the donor cells to produce sEVs with altered expression of specific cargos. Additionally, sEVs could potentially be loaded with anti-cancer drugs and magnetic particles simultaneously, and the sEVs could be attracted to the tumor site by the application of a magnetic field. Apart from acting as vehicles to deliver drugs specifically and effectively, sEVs can be used as an adjuvant to modulate the immune response or as cancer vaccines to potentially prevent tumor early progression.

Currently, EVs derived from immune cells (imEVs) or MSCs are being explored for their potential in cancer therapy. imEVs inherit the intrinsic features of their donor cells regarding antigen presenting and cytotoxic functions. Either original or manipulated imEVs can be used for drug delivery or immunoadjuvant with high specificity, efficiency, and biosafety. DC-sEVs are the most well-studied imEVs and are earliest to enter clinical trials due to their low immunogenicity and innate capacity for inflammation-directed tumor accumulation. Clinical trials utilizing DC-sEV vaccines have demonstrated the safety and potential for treatment of advanced non-small cell lung cancer, melanoma, and colorectal cancer (102). Furthermore, cargo loading enhances the immunogenicity of DCs, making personalized immunotherapy a promising approach for eradicating heterogeneous tumors. For instance, a study utilized an HCC-targeting peptide (P47-P), an AFP epitope (AFP212-A2), and a functional domain of high mobility group nucleosome-binding protein 1 (N1ND-N) as exosomal anchor peptides to create a “trigger” DC sEV vaccine (DEXP&A2&N) (103). This tailored approach induces an immune response based on individual patients and can serve as a method for personalized immunotherapy of HCC. Interestingly, a study demonstrated that DCs loaded with the tumor derived exosomes (TEX) could promote T cell proliferation and enhance the release of multiple cytokines, including IFN-γ, IL-2, TNF-α, IL-10, and TGF-β. However, the DC-TEX also elevated the population of PD1^+^CD8^+^T cells (48). In addition, DCs co-cultured with plasma sEVs from HCC patients showed higher levels of IL-2 and IL-12 and could activate T cells. These DC-TEX also showed antitumor effects in subcutaneous tumors (104).

## 6 Challenges, future directions, and conclusions

sEVs from cancer, immune, and stromal cells have been shown to be capable of promoting or suppressing HCC stemness by modulating various signaling pathways. Understanding the cargos and functions of those sEVs can provide valuable insights into the development of novel diagnostic or therapeutic approaches targeting CSCs.

sEVs have shown diagnostic and therapeutic implications in clinical settings (105). For the clinical use of sEVs as biomarkers, a major challenge lies in the lack of standardized methods for sEV isolation. Ultracentrifugation is recognized as the gold standard and most commonly used method for sEV isolation due to its high purity. However, it is laborious and time-consuming, and the yield is relatively poor. Polymer-based precipitation is a quick and easy method with high yields of sEVs. However, it can also co-precipitate non-vesicular components, leading to poor purity. In recent years, immunoaffinity capture has emerged as a highly specific and sensitive method that can isolate sEVs based on specific markers. However, it requires specialized antibodies or aptamers, which can be expensive, and may not be suitable for large quantities of samples. In addition, microfluidic techniques have gained significant interest in EV-based diagnostics, but there is a need to bridge the gap between lab-on-chip techniques and current standards in EV characterization and bioproduction. Scaling up devices for high-volume EV bioproduction and ensuring simplicity, reproducibility, and transferability of technology are crucial (106). Furthermore, defining the precise source of sEVs in plasma is challenging as they originate from various blood cells and multiple organs, thus the analysis can be complicated. Since most of the plasma sEVs are derived from peripheral blood cells, depletion of blood cell-sEVs from the pool of sEVs is a strategy to uncover candidates derived from tumor cells. Furthermore, apart from the altered expression levels of some candidate cargos in sEVs, more attention should be paid to some genetic, translational, or post-translational modifications that may be upregulated or uniquely detected in plasma sEVs.

In addition to manipulation of sEVs, intervention of EV biogenesis is also a therapeutic method. Neutral sphingomyelinase 1 (NSMase1), an enzyme essential in the exosome biogenesis pathway, is downregulated in HCC tissues. NSMase1 enrichment in the exosomes was found to disrupt the ceramide/sphingomyelin balance and thus induce apoptosis within the recipient HCC cells (107). SNARE is a highly conserved protein that is responsible for the vesicle fusion, and manipulation or inhibition of some fusogenic proteins, such as the synaptotagmin (Syt) and Munc13, have significant implications in treating cancers (108). Therefore, developing therapeutic strategies that specifically target the exosome synthesis pathways, particularly those crucial for tumor cells, may be a promising direction for further research.

Currently, the functions of imEVs have been relatively understudied in HCC. Further exploration of the crosstalk between sEVs and the immune system in the context of HCC stemness is necessary. We also need to be aware that some cargos from plasma sEVs could promote HCC progression through regulating immune cells. For example, LncRNA FAL1 was upregulated in the blood exosomes in HCC patients and was able to induce polarization of macrophage into pro-tumorigenic M2, which resulted in higher proliferation and upregulated β-catenin signaling in HCC cells (109). However, targeting the interaction of sEVs and immune cells may potentially alter the immune response to effectively combat tumors.

DC-sEVs stimulated by tumor antigen exhibit higher cytolytic rate and induced T cell activity. In HCC, it was found that the combination of DC-sEVs with microwave ablation significantly inhibited tumor growth as compared with microwave ablation monotherapy, with increased CD8^+^T cells and fewer Treg cells in tumor sites (110). Although promising results have been observed in preclinical trials in some cancer types, the clinical application of DC- sEVs faces a primary challenge in terms of their limited efficacy in activating T cells.

There are several ongoing clinical trials registered on the ClinicalTrials.gov website that investigate the use of EVs as therapeutic interventions for head and neck cancer (NCT03109873, NCT01668849), colon cancer (NCT01294072), and metastatic pancreatic cancer with KRAS G12D mutation (NCT03608631). However, currently there are no ongoing clinical trials using sEVs for HCC treatment. The clinical applications of sEVs are still in their early stages, and the most therapeutic research using sEVs is currently being done with DCs and MSCs. Although sEVs have shown advantages over traditional therapeutic methods, such as increased biosafety and selectivity, the main hurdles for their translation to clinical use are the high impurities, low yields, insufficient anticancer effects *in vivo*, and concerns around stability and toxicity. Despite these challenges, sEVs hold great promise for future clinical therapies for fighting cancer. Utilizing sEVs as therapeutic agents for HCC and other cancers is a promising avenue for future research. Development of methods to selectively target and eliminate CSCs while sparing normal stem cells is much awaited.

## Author contributions

LT: Conceptualization, Formal analysis, Funding acquisition, Resources, Visualization, Writing – original draft. JL: Conceptualization, Formal analysis, Resources, Visualization, Writing – original draft. IN: Conceptualization, Formal analysis, Resources, Visualization, Funding acquisition, Project administration, Supervision, Writing – review & editing.
